# Functional and phenotypic heterogeneity of group 3 innate lymphoid cells

**DOI:** 10.1111/imm.12697

**Published:** 2017-01-03

**Authors:** Felipe Melo‐Gonzalez, Matthew R. Hepworth

**Affiliations:** ^1^ Faculty of BiologyMedicine and HealthSchool of Biological SciencesDivision of Infection, Immunity and Respiratory MedicineUniversity of ManchesterManchesterUK

**Keywords:** group 3 innate lymphoid cells, innate lymphoid cell, lymphoid tissue‐inducer cells, mucosal immunology

## Abstract

Group 3 innate lymphoid cells (ILC3), defined by expression of the transcription factor retinoid‐related orphan receptor *γ*t, play key roles in the regulation of inflammation and immunity in the gastrointestinal tract and associated lymphoid tissues. ILC3 consist largely of two major subsets, NCR ^+^ ILC3 and LTi‐like ILC3, but also demonstrate significant plasticity and heterogeneity. Recent advances have begun to dissect the relationship between ILC3 subsets and to define distinct functional states within the intestinal tissue microenvironment. In this review we discuss the ever‐expanding roles of ILC3 in the context of intestinal homeostasis, infection and inflammation – with a focus on comparing and contrasting the relative contributions of ILC3 subsets.

**Abbreviations**
CHILP: common helper ILC progenitor
GM‐CSF: granulocyte–macrophage colony‐stimulating factor
ID2: inhibitor of DNA‐binding protein
IFN‐*γ*: interferon‐*γ*
IL: interleukin
ILC: innate lymphoid cell
ILCP: ILC progenitor
ILF: isolated lymphoid follicule
LTi: lymphoid tissue‐inducer
NCR^+^: natural‐cytotoxicity‐receptor‐positive
NK cell: natural killer cell
ROR*γ*t: retinoid‐related orphan receptor *γ*t
SFB: segmented filamentous bacteria
Th: T helper
TNF‐*α*: tumour necrosis factor‐*α*

## The innate lymphoid cell family

The innate lymphoid cell (ILC) family has been the focus of intense investigation over recent years and ILC have emerged as key players in immune responses within multiple organs, particularly barrier surfaces such as the lung, skin and gastrointestinal tract.^1^, ^2^, ^3^, ^4^ The current nomenclature broadly divides ILC into three subgroups that are defined by their master transcription factor usage and cytokine‐producing capacity, and which closely mirror CD4^+^ T helper (Th) cell subsets.^3^ However, in contrast to T cells, ILC lack antigen‐specific receptors and do not require *de novo* proliferation and polarization for cytokine production, instead acting as ‘pre‐primed’ terminal effector cells that constitutively reside in barrier tissues and respond to alarmins and cytokine signals released following tissue damage.

Group 1 ILC (ILC1), which include classical natural killer (NK) cells as well as *bona fide* non‐NK ILC1, respond to interleukin‐12 (IL‐12), IL‐15 and IL‐18 and are defined by their expression of the transcription factor T‐bet, production of the cytokines interferon‐*γ* (IFN‐*γ*) and tumour necrosis factor‐*α* and mediate immune responses to intracellular pathogens and tumours.^3^, ^5^, ^6^, ^7^ Group 2 ILC (ILC2) respond to tissue‐derived alarmins including IL‐25 and IL‐33, are characterized by their expression of GATA‐3, produce the cytokines IL‐5, IL‐9 and IL‐13 and contribute to immune responses against multicellular pathogens (such as nematode parasites) as well as the pathogenesis of type 2 inflammatory diseases including asthma and atopic dermatitis.^1^, ^2^, ^3^

Retinoid‐related orphan receptor *γ*t (ROR*γ*t) ‐expressing group 3 ILC (ILC3) respond to signals from tissue‐resident myeloid cells, such as IL‐1*β* and IL‐23, by producing cytokines including IL‐17A, IL‐17F and IL‐22.^1^, ^2^, ^3^, ^4^, ^8^ In the adult mouse, ILC3 can be further subdivided into at least two major subsets – the first is defined by the expression of the NK cell‐associated receptor NKp46 and has been termed natural‐cytotoxicity‐receptor‐positive ILC3 (NCR^+^ ILC3). The second major subset is defined by their expression of the chemokine receptor CCR6 and phenotypically mirrors fetal lymphoid tissue‐inducer cells (LTi), and so have been termed LTi‐like ILC3. Despite both subsets sharing significant functional overlap, increasing evidence suggests that these subsets also have distinct, non‐redundant functions.

## Group 3 innate lymphoid cell development

**Figure 1:**
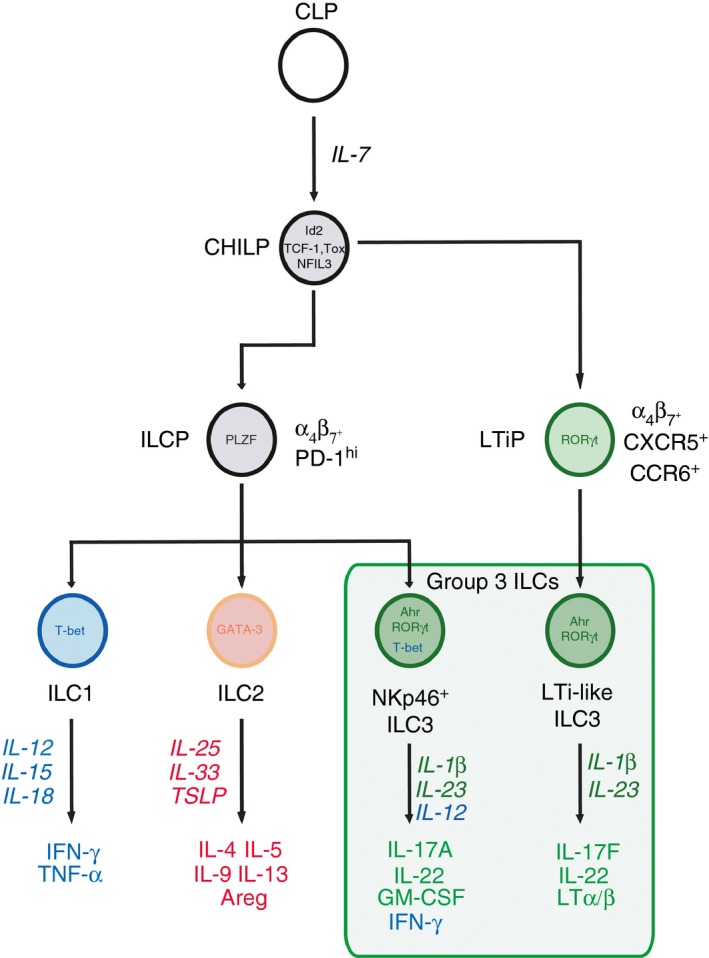
Ontogeny of group 3 innate lymphoid cells (ILC3). All ILC subsets are derived from a common lymphoid progenitor (CLP), Initial commitment towards an ILC fate is associated with up‐regulation of the transcription factor inhibitor of DNA‐binding protein (ID2) and is dependent upon interleukin‐7 (IL‐7). This progenitor population gives rise to all IL‐7receptor (IL‐7R) expressing ILC family members and has been termed the common ‘helper’ ILC progenitor (CHILP). Recent evidence suggests that bifurcation of progenitors occurs downstream of the CHILP. Acquisition of promyelocytic leukaemia zinc finger (PLZF) expression and expression of programmed cell death protein‐1 (PD‐1) mark further commitment to the ILC lineage (ILCP). However, while ILCP give rise to ILC1, ILC2 and NKp46^+^ ILC3s, these cells fail to generate lymphoid tissue‐inducer (LTi)‐like ILC3 progeny. In contrast, a distinct LTi‐precursor (LTiP) lacking PLZF‐expression is defined as CXCR5^+^ *α*_4_ *β*_7_^+^ and CCR6^+^ and gives rise to fetal LTi and adult LTi‐like ILC3. The bifurcation of group 3 ILC subsets during development may imprint differential transcriptional programmes that underlie the function of mature ILC3 subsets in the periphery.

All ILC subsets (ILC1, ILC2 and ILC3), as well as adaptive lymphoid cells (B and T cells), are derived from a common lymphoid precursor (CLP) that is present in the fetal liver and adult bone marrow.^9^, ^10^ Recent studies have elegantly defined the presence of common ILC precursors that develop downstream of the common lymphoid precursor, and which have lost the potential to develop into T and B cells.^5^, ^11^, ^12^, ^13^ The initial differentiation of ILC‐restricted progenitors from multipotent common lymphoid precursors is dependent upon IL‐7 and characterized by expression of the inhibitor of DNA‐binding protein (ID2)^14^, ^15^ (Fig. 1). ID2^+^ progenitors give rise to all IL‐7R*α*‐expressing ILC and have been termed the ‘common helper ILC progenitor’ (CHILP) to distinguish these cells from NK cell progenitors^5^ (Fig. 1). Progressive commitment to the ILC lineage is also dependent, to varying degrees, on a multitude of transcription factors including GATA‐3, TOX, TCF‐1 and NFIL3^16^, ^17^, ^18^, ^19^, ^20^, ^21^ (and reviewed in detail in references 10 and 22).

Further commitment towards individual ILC subgroups is associated with transition through a phase characterized by promyelocytic leukaemia zinc finger (PLZF) expression (encoded by *Zbtb16*) and high surface expression of programmed cell death protein‐1 (PD‐1) (Fig. 1).^11^, ^23^ Whereas ID2^+^ CHILP give rise to all major ILC subsets, PLZF‐expressing progenitors (ILCP) gave rise to ILC1, ILC2 and NKp46^+^ ILC3 – but not LTi‐like ILC3 – suggesting that PLZF^+^ ILCP have lost the capacity to generate LTi‐like ILC3 progeny.^11^ In line with this finding, a bifurcation of ILC progenitor programming occurs before acquisition of PLZF expression that generates a distinct LTi precursor that is Id2^+^ PLZF^−^ and characterized by the expression of CXCR5, *α*_4_ *β*_7_ and CCR6 (Fig. 1).^12^, ^13^ These studies suggest that adult LTi‐like ILC3 may be developmentally divergent from other ILC, including NKp46^+^ ILC3, and indicate that these two ILC3 subsets may differ to a greater degree than currently appreciated.

Expression of ROR*γ*t is essential for the development and maturation of all members of the ILC3 family, including fetal LTi, adult LTi‐like ILC3 and NKp46^+^ ILC3.^24^, ^25^, ^26^, ^27^ ROR*γ*t‐expressing progenitors are present in the fetal liver of mice.^14^ Although much of ILC3 development is thought to take place in this compartment before birth, ILC3 may also develop from adult bone marrow ILCP and immature committed ILC3 precursors have the capacity to migrate from the bone marrow to the periphery where they undergo final maturation.^14^, ^28^, ^29^, ^30^ Similarly, human adult CD34^+^ haematopoietic precursor cells have been described that exhibit ROR*γ*t expression and an ILC3 transcriptional signature,^29^ while human CD34^+^ ROR*γ*t^+^ precursors have been found in adult tonsils and the gut.^28^, ^29^, ^31^ Hence, in addition to the generation of fully committed mature ILC3 subsets during fetal development, further ILC3 maturation of precursors may occur locally in the tissue following birth.

## Tissue‐resident ILC3: from cradle to grave

Group 3 ILC are among the first immune cells to seed intestinal tissue. Before birth, fetal LTi‐like ILC3 are crucial initiators of peripheral secondary lymphoid tissue organogenesis and, in concert with endothelial lymphoid tissue organizer populations,^32^ are required for the generation of lymphoid tissues, including the mesenteric lymph node and Peyer's patches in the small intestine^24^, ^33^ (reviewed in detail in ref. 34). LTi act to orchestrate the generation of nascent lymphoid tissue through a process that requires the interaction between LTi cells and stromal organizer cells and that is dependent upon LTi‐expressed lymphotoxin.^34^, ^35^

Similarly, interactions between adult LTi‐like ILC3 and stromal cells are also important for lymphoid tissue formation later in life, as evident from the formation of discrete LTi‐like ILC3 clusters termed cryptopatches in the intestinal lamina propria that form 1–2 weeks after birth.^34^, ^36^ Cryptopatches predominantly contain LTi‐like ILC3 and dendritic cells organized around stromal cell populations, and are situated close to the bottom of intestinal crypt structures. Cryptopatch‐resident ILC3 maintain the local crypt stem cell pool and regulate tissue repair following inflammation, chemotherapy or transplantation.^37^, ^38^, ^39^ In addition, cryptopatches act as a site for B‐cell recruitment to form mature tertiary lymphoid structures known as isolated lymphoid follicules (ILFs).^36^, ^40^, ^41^ ILFs are important sites for production of IgA against intestinal bacteria and ILF hyperplasia occurs in the context of inflammation or bacterial outgrowth,^41^ suggesting that ILFs are ILC3‐regulated inducible lymphoid tissues required for orchestration of local responses to commensal bacteria.

Commensal microbiota‐derived signals are critical in driving ILC3 recruitment and expansion in the intestine.^27^, ^30^ Before birth, ILC3 seeding of the fetal intestine may be modulated by passively transmitted maternal signals, including those derived from the microbiota.^42^ Aryl hydrocarbon receptor (Ahr) ligands bound by maternal antibodies cross the placenta and increase the number of NKp46^+^ ILC3 in the uncolonized fetal intestine.^42^ Additional environmental signals experienced *in utero* can further modulate ILC3. Similarly, maternal retinoids were found to be critical for the development of LTi cells in the developing fetus by regulating transcription of ROR*γ*t.^43^ Dietary metabolites also play key roles in supporting ILC3 function and stability. Vitamin A deprivation results in a loss of intestinal ILC3 in adult mice, which can be reversed upon supplementation of diet with the vitamin A‐derived metabolite retinoic acid.^44^, ^45^ Similarly, Ahr acts to sense soluble aromatic hydrocarbons – present in the diet and produced by commensal bacteria – and is essential for the development of both LTi‐like and NKp46^+^ ILC3, as well as IL‐22 production.^14^, ^27^, ^28^, ^46^, ^47^, ^48^, ^49^, ^50^, ^51^ Together these signals tune ILC3 numbers and responses and establish bidirectional interactions between ILC3 and the commensal microbiota during the initial colonization of barrier tissue sites.

In contrast, relatively little is known about the fate or lifespan of ILC3 subsets. ILC3 have a long half‐life in tissues and lymph nodes and are largely replenished locally within the tissue.^30^, ^52^ Surprisingly, ILC3 can maintain their phenotype and function even in the absence of ROR*γ*t expression and persist for a significant length of time.^53^ Nonetheless, changes in dietary metabolites, chronic inflammation or infectious insult result in disruption of ILC3 effector functions or even irreversible depletion of ILC from the intestinal tissue altogether.^54^, ^55^ Further studies are required to understand how ILC3 numbers and functions are maintained and how they can be restored following damage or infection‐induced perturbation.

## ILC3 plasticity and heterogeneity

**Figure 2:**
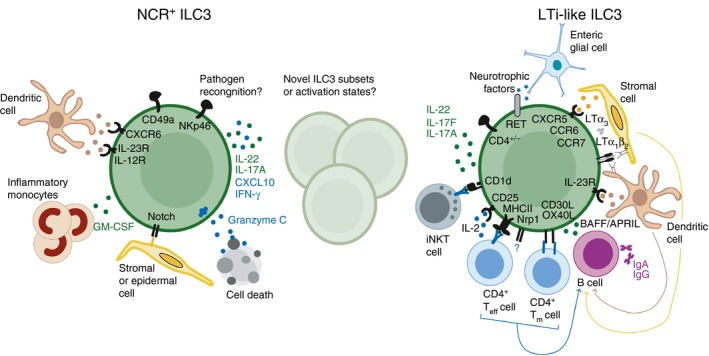
Group 3 innate lymphoid cell (ILC3) subset‐defining phenotypes and functions. Fully mature ILC3 subsets in peripheral tissues are broadly categorized into two major subsets; NKp46^+^ ILC3 (left panel) and lymphoid tissue‐inducer (LTi) ‐like ILC3 (right panel). Although both subsets express retinoid‐related orphan receptor *γ*t (ROR *γ*t) and share some core functions, emerging evidence suggests subset‐restricted functions. Phenotypic surface markers that typically used to define ILC3 subsets (bold) include natural killer (NK) ‐cell‐associated receptors, such as NKp46 and CD49a, for natural‐cytotoxicity‐receptor‐positive (NCR ^+^) ILC3s, whereas LTi‐like ILC3s are primarily characterized by their expression of CCR6 and variable expression of CD4. Differential localization of NKp46^+^ ILC3 and LTi‐like ILC3 to the lamina propria and lymphoid tissues, respectively, is determined by differential chemokine receptor profiles. The difference in ILC3 subset localization is indicative of functional specialization. LTi‐like ILC3 express multiple molecules that endow this subset with the ability to indirectly and directly modulate innate‐like and adaptive immune responses in lymphoid tissues. In contrast, NCR ^+^ ILC3 appear to largely lack the ability to modulate adaptive immune responses but exhibit unique interactions with populations of myeloid and stromal cells in the tissue that drive the relatively pro‐inflammatory phenotype of this subset. Finally, although the majority of mature ILC3s exhibit NCR ^+^ or LTi‐like phenotypes recent transcriptomic studies indicate that multiple intermediate activation states may exist, which are likely determined by transition through metabolic states and differing environmental niches.

Although the developmental networks that drive ILC3 ontogeny have been extensively defined in recent years, the degree to which mature ILC3 subsets differ transcriptionally and functionally in peripheral tissues is only now beginning to emerge. Recent high‐resolution epigenetic and transcriptomic analyses of human and murine ILC3 have begun to clarify subset‐specific functional and transcriptional phenotypes, while revealing that ILC3 exhibit a higher degree of plasticity and heterogeneity than previously appreciated.^56^, ^57^, ^58^, ^59^, ^60^ Despite sharing many overlapping transcriptional pathways LTi‐like and NKp46^+^ ILC3 subsets exhibit considerable differences in chromatin accessibility and predicted transcription factor occupancy among accessible gene regulatory elements.^58^, ^59^, ^60^ Differences between these two canonical ILC3 subsets are further highlighted on the transcriptional level.^56^, ^60^ Despite significant overlap in expression of core ILC3 member genes, including *Rorc* and *Il23r*, NKp46^+^ ILC3 and LTi‐like ILC3 also displayed subset‐restricted gene expression^56^ (Fig. 2). NKp46^+^ ILC3 are characterized by subset‐restricted expression of multiple genes required for their development and maturation, such as *Il12rb2*,* Tbx21* and *Notch1*,^51^, ^61^ and are defined by expression of eponymous NK cell‐associated receptors. Recent evidence suggests that NKp46 expression may act as a novel pattern recognition molecule to facilitate responses to fungal pathogens,^62^ whereas engagement of NKp44 on human ILC3 results in pro‐inflammatory cytokine production.^63^ Although NCR^+^ ILC3 are largely thought to be non‐cytotoxic cells, NKp46^+^ ILC3 express *Gzmc* – which encodes a murine granzyme molecule known to induce cell death.^60^, ^64^ This observation is in line with a considerable transcriptional overlap between NKp46^+^ ILC3 and ILC1, which also exhibit a degree of cytotoxic capacity.^65^ Indeed NKp46^+^ ILC3 were found to share more transcripts with ILC1s than with LTi‐like ILC3.^56^

The LTi‐like ILC3 demonstrate subset‐specific expression of characteristic genes such as *Cd4* and *Ccr6*, but also express additional genes indicative of novel functionality in LTi‐like ILC3 (Fig. 2). Among these is the gene encoding RET – a neuroregulatory receptor – which is important for LTi‐mediated formation of Peyer's patches.^66^ LTi‐like ILC3 expression of RET endows them with the ability to respond to neurotrophic factors released from enteric glial cells in response to microbial signals. Neurotrophic factor signalling reinforced production of IL‐22 and modulation of epithelial barrier integrity,^67^ suggesting that LTi‐like ILC3 may have the unique potential to respond to environmental cues from the enteric nervous system. Indeed, inflammation‐induced tertiary lymphoid tissue formation – which is regulated by ILC3 – requires vagal nerve innervation in the intestine.^68^ In addition, LTi‐like ILC3 were also found to specifically express Neuropilin‐1, an immunoregulatory factor that is expressed by subsets of regulatory T cells,^56^, ^69^, ^70^ which may indicate additional novel and subset‐restricted roles for LTi‐like ILC3.

**Figure 3:**
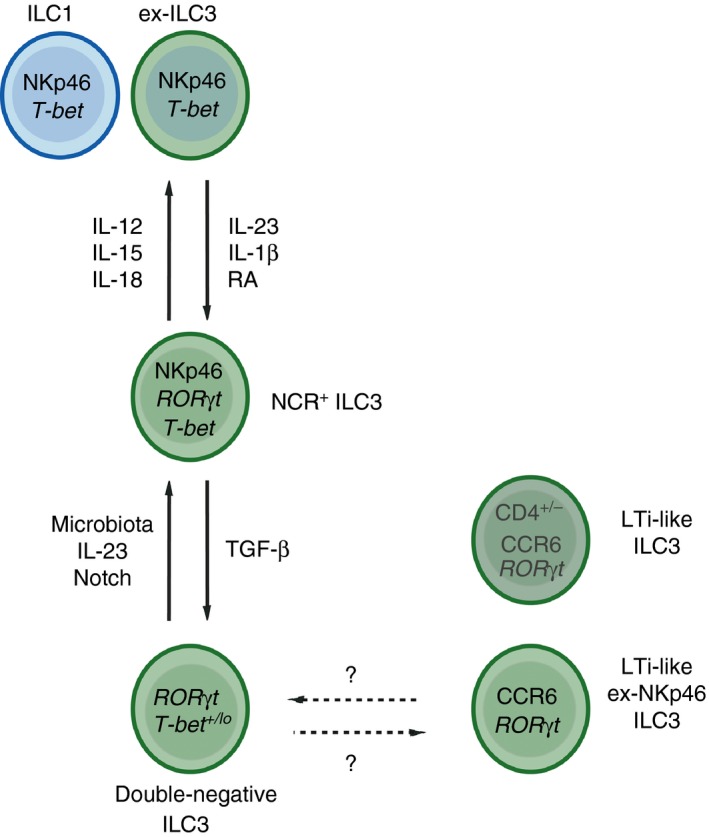
Plasticity and heterogeneity of intestinal group 3 innate lymphoid cell (ILC3). In addition to NKp46^+^ ILC3 and CCR6^+^ lymphoid tissue‐inducer (LTi) ‐like ILC3 a ‘double‐negative’ NKp46^−^ CCR6^−^ ILC3 population is present in the intestine. These cells act as a plastic precursor pool that gives rise to NKp46^+^ ILC3s in a reversible manner in response to local signals, which is regulated by a gradient of T‐bet expression. In addition, NKp46^+^ ILC3s may further lose retinoid‐related orphan receptor *γ*t (ROR *γ*t) expression in response to inflammatory cytokines and take on a phenotype that mirrors ILC1. These ‘ex‐ILC3’ retain their plasticity and are able to revert to an NKp46^+^ ILC3 or ‘double‐negative’ precursor phenotype upon alterations in the local cytokine milieu. LTi‐like ILC3 are thought to develop from a distinct progenitor. However, a small proportion of CCR6^+^ ILC3 display a history of NKp46 expression (ex‐NKp46 ILC3), suggesting that they may also be derived from tissue‐resident plastic ILC3s, although these cells largely lack expression of other LTi‐associated markers such as CD4 and MHCII. These findings blur the boundaries of ILC3 subset ontogeny and necessitate further studies to determine the relationship between *bona fide* LTi‐like ILC3 and those derived from plastic precursors. The high degree of reversible plasticity among ILC3s suggests a highly dynamic regulation of phenotype and function in response to environmental and infectious cues. This may be beneficial and allow transient increases in ILC3s with a pro‐inflammatory capacity when required, while allowing reversion to a more tolerogenic balance of ILC3 subsets following resolution of the infection or inflammatory insult.

Despite the identification of ILC3 subset‐specific functions, recent evidence has also highlighted a significant degree of heterogeneity among tissue‐resident ILC3, which blurs the boundaries of current subset classification. Single cell‐sequencing analyses of all ILC present in the small intestinal lamina propria of mice distinguished five discrete transcriptional states among ILC3.^60^ Several transcripts identified have not previously been associated with a function in ILC3 and encode genes involved in metabolism, as well as chemokines and growth factors (Fig. 2 and^60^). Nonetheless, comparison mapping of bulk sequencing data of sorted NKp46^+^ ILC3 and LTi‐like ILC3 subsets demonstrated that the two major ILC3 subsets span several transcriptional states, suggesting a dynamic interchange between transcriptional or functional states in response to environmental cues and/or cellular interactions.^60^ This is in line with the finding that intestinal ROR*γ*t‐expressing ILC demonstrate significant plasticity (Fig. 3). In addition to CCR6^+^ LTi‐like ILC3 and NKp46^+^ ILC3, the small intestine contains a population of ‘double‐negative’ ROR*γ*t^+^ CCR6^−^ NKp46^−^ ILC3.^61^, ^71^ ‘Double‐negative’ ILC3 represent intestinal precursors of the NKp46^+^ ILC3 subset, and acquire NKp46 and IFN‐*γ* expression in a process driven by up‐regulation of T‐bet and dependent upon IL‐23, Notch signalling and the microbiota.^51^, ^61^, ^71^ In addition, fate‐mapping studies revealed the presence of NKp46^+^ T‐bet^+^ ‘ex‐ILC3’ that had lost ROR*γ*t‐expression in response to pro‐inflammatory cytokine stimulation and that functionally and phenotypically resemble ILC1 5, 61, 71 (Fig. 3). Complimentary findings in human tissue samples demonstrated reversible conversion between NCR^+^ ILC3 subsets and ILC1‐like ‘ex‐ILC3’ dependent on the local cytokine milieu, which was determined by tissue‐resident myeloid populations.^72^, ^73^ These findings demonstrate significant plasticity among CCR6^−^ ILC3, which is both tuneable and reversible. However, the relationship of this plastic ILC3 lineage to LTi‐like ILC3 is unclear.

Although CCR6^+^ LTi‐like ILC3 are thought to be relatively stable and ontologically distinct, recent evidence suggests that a proportion of ILC3 expressing a CCR6^+^ LTi‐like phenotype may also be derived from the plastic population of ILC3 in the intestine. Fate‐mapping of the NKp46 locus revealed populations of ‘ex‐NKp46 ILC3’ that no longer expressed NKp46, many of which had reverted to a ‘double‐negative’ ILC3 phenotype.^74^, ^75^ Transition between phenotypes was regulated locally via Notch and suppressed by transforming growth factor‐*β*, suggesting that the NKp46^+^ ILC3 phenotype is unstable and dynamically regulated.^74^, ^75^ Surprisingly, a small proportion of CD4^−^ CCR6^+^ ILC3 also exhibited a history of NKp46 expression, suggesting that at least some ILC3 with an LTi‐like phenotype may be derived from the same precursor as NKp46^+^ ILC3, rather than from a pure LTi‐precursor.^74^ Taken together, these findings highlight the heterogeneity and plasticity of ILC3 in the intestine, challenge the distinction between mature ILC3 subsets and highlight the difficulty of categorizing ILC3 subsets based on a limited numbers of surface markers.

In humans it has been challenging to address the full extent of ILC3 heterogeneity and to identify human ILC3 subsets that are directly analogous to murine LTi‐like ILC3 and NKp46^+^ ILC3 subsets. Recent single‐cell sequencing studies using non‐biased clustering of transcriptome data among human ILC identified three distinct ROR*γ*t‐expressing ILC3 subsets.^57^ Although all human ILC3 subsets shared expression of *Rorc*,* Tox*,* Ahr*,* Tcf7* and Notch‐induced genes, distinct subsets could be distinguished by the expression of either NKp44, HLA‐associated genes or *SELL* (which encodes CD62L).^57^ The latter subset also expressed CD45RA and responded poorly to re‐stimulation, suggesting the presence of a ‘naive’ or undifferentiated ILC3 phenotype in humans. Interestingly, murine ‘ex‐NKp46 ILC3’ that have reverted to a ‘double‐negative’ phenotype also demonstrate relatively suppressed cytokine‐producing capacity.^74^ Hence, further investigation is required to determine the relationship of this ‘naive’ human ILC3 subset to other established human ILC3 phenotypes, particularly whether this population is analogous to the plastic ‘double‐negative’ ILC3 precursor found in mice.

Both mouse and human single‐cell sequencing approaches highlighted expression of MHCII as a defining marker of a distinct ILC3 subset.^56^, ^60^ MHCII has previously been identified on adult LTi‐like ILC3 in mice and humans,^76^ suggesting that *bona fide* LTi‐like ILC3 may be distinguished functionally from the plastic pool of CCR6^−^ ILC3 (including NCR^+^ ILC3) by their ability to interact with adaptive immune cells. Hence, it is tempting to speculate that human NKp44^+^ and HLA^+^ ILC3 subsets may be analogous to murine NKp46^+^ and MHCII^+^ LTi‐like ILC3 populations, respectively – although further studies are required to define these populations further in multiple human tissues.

## ILC3 functions under homeostatic conditions

ILC3 play critical roles in maintaining intestinal tissue health and homeostasis, predominantly through the regulation of steady‐state interactions with commensal bacteria. This is achieved by constitutive production of cytokines that act to regulate epithelial barrier function and commensal bacterial diversity, as well as through interactions with cells of the adaptive immune system that further enforce a tolerogenic environment to prevent commensal bacteria‐driven inflammation. Emerging evidence suggests that ILC3 subsets may differentially contribute to homeostasis in a subset‐specific manner.

Steady‐state ILC3 are the dominant source of IL‐22, a cytokine that acts on non‐haematopoietic cells including intestinal epithelial cells.^8^ IL‐22 signalling induces a cascade of gene expression in epithelial cells typified by the secretion of antimicrobial peptides such as RegIII proteins, S100a8 and S100a9.^8^, ^77^ These antimicrobial peptides act to regulate spatial segregation of commensal bacteria from the epithelial barrier by establishing a gradient of bactericidal activity, which has been termed the ‘demilitarized zone’.^77^ Production of IL‐22 further reinforces spatial segregation of commensal bacteria by driving transcription of membrane‐bound mucins and goblet cell hyperplasia.^78^, ^79^

Mice with disrupted ILC3‐derived IL‐22 responses exhibit altered gut microbiota and increased susceptibility to experimentally induced colitis.^49^, ^80^, ^81^, ^82^ Most notably, expansion of segmented filamentous bacteria (SFB) – a canonical Th17‐inducing commensal species – occurs in the absence of ILC3 responses.^49^ ILC3‐derived IL‐22 induced by SFB colonization stimulates serum amyloid protein production from epithelial cells that drive the recruitment of Th17 cells to the ileum, which subsequently act to control SFB burdens.^83^ Both intestinal NKp46^+^ ILC3 and LTi‐like ILC3 produce IL‐22 but inhabit distinct niches within the lamina propria and intestinal lymphoid structures. Interestingly, mice specifically lacking NKp46^+^ ILC3 do not demonstrate dysbiosis, outgrowth of SFB or changes in Th17 cell numbers in the small intestine at steady state.^84^ Further studies specifically depleting LTi‐like ILC3 are needed to determine whether these findings merely reflect redundancy among IL‐22‐producing ILC3 or subset‐specific roles in the regulation of commensal bacterial species. ILC3‐derived IL‐22 also results in fucosylation of intestinal epithelial cell‐associated carbohydrate chains following colonization of the intestinal tract by commensal bacteria.^85^, ^86^, ^87^ Whereas, certain species of mutualistic bacteria possess the necessary machinery to use carbohydrate fucose as an energy source, many pathogenic bacteria lack this ability and are outcompeted for energy sources. IL‐22‐dependent fucosylation favours the occupancy of mutualistic species in intestinal niches close to the epithelial barrier and was shown to protect against infections by *Salmonella* spp., *Citrobacter rodentium* and the opportunistic pathogen *Enterococcus faecalis*.^85^, ^86^, ^87^

In addition, ILC3‐derived IL‐22 is essential to prevent bacterial translocation and infections with opportunistic pathogens present in the commensal flora. Mice lacking homoeostatic IL‐22 production or depleted for ILC3 demonstrate bacterial translocation to the peripheral organs as a result of disrupted barrier integrity and a failure to contain lymphoid tissue‐resident commensal bacteria species.^88^, ^89^, ^90^ Thus, ILC3 play critical roles during homeostasis by regulating epithelial barrier responses and the balance of commensal bacterial species, largely through the production of IL‐22.

## ILC3 interactions with adaptive immunity

NKp46^+^ ILC3 are largely dispersed in the intestinal lamina propria, whereas LTi‐like ILC3 are found clustered within lymphoid tissues where they have the potential to influence the adaptive immune system. This localization is determined in part through subset‐specific chemokine receptor expression. Localization of NKp46^+^ ILC3 is dictated by expression of CXCR6 resulting in retention in the lamina propria in response to CXCL16‐producing dendritic cell subsets.^91^, ^92^ LTi‐like ILC3 localize to secondary and tertiary lymphoid tissues driven by expression of CXCR5, CCR6 and CCR7 and cluster within interfollicular regions where they are ideally poised to interact with both T and B cells. Indeed, LTi‐like ILC3 express multiple proteins that enable interactions with adaptive immune cells^93^, ^94^ (Fig. 2).

MHCII is highly expressed on CCR6^+^ LTi‐like ILC3 resident in lymph nodes, but absent on NKp46^+^ ILC3.^55^, ^76^, ^94^, ^95^ Under homeostatic conditions MHCII^+^ ILC3 act to limit intestinal inflammation by deletion of commensal bacteria‐specific CD4^+^ T cells from the intestine and associated lymphoid tissues.^55^, ^76^, ^96^ This pathway may have implications for human disease as HLA‐DR expression on human intestinal ILC3 is reduced in paediatric Crohn's disease patients.^55^ MHCII^+^ ILC3 migrate from the intestine to the mesenteric lymph node in a CCR7‐dependent manner, suggesting that antigens may be acquired locally in the tissue before migration to lymph nodes.^94^ Surprisingly, a dichotomous relationship between MHCII and IL‐22 expression was recently reported among intestinal ILC3,^60^ seemingly in contrast to previous reports that LTi‐like ILC3 are a dominant source of IL‐22 of the intestine^56^, ^67^, ^97^ (Fig. 2). Hence, further investigation is required to determine whether LTi‐like ILC3‐intrinsic expression of MHCII and IL‐22 is mutually exclusive and/or determined by the local tissue environment.

In contrast to canonical antigen‐presenting cells MHCII^+^ LTi‐like ILC3 lack expression of the classical co‐stimulatory molecules CD80 and CD86 at steady state, in line with an inhibitory role for ILC3‐mediated antigen presentation.^55^, ^76^ However, under inflammatory conditions MHCII^+^ ILC3 can up‐regulate these molecules and act to promote immune responses.^95^ In addition, ILC3 can augment CD4^+^ T‐cell responses through expression of auxiliary co‐stimulatory molecules. ILC3 promote the survival of memory CD4^+^ T cells and aid T cell‐dependent antibody responses by expression of OX40L and CD30L.^98^, ^99^, ^100^ Interactions between ILC3 and adaptive immune populations are probably also bi‐directional as mice lacking T cells demonstrate increased intestinal ILC3 numbers and IL‐22 production.^101^ Furthermore, a recent study demonstrated CCR6^+^ LTi‐like ILC3 express the lipid antigen‐presenting molecule CD1d (Fig. 2), and process and present lipids to activate invariant NK T cells, whereas CD1d‐dependent cross‐talk also induced IL‐22 production from the presenting ILC3.^102^ In addition, ILC3 prevent spontaneous CD8^+^ T‐cell activation in neonatal lymph nodes, although it remains unclear whether modulation of CD8^+^ T‐cell responses is also dependent upon ILC3 antigen‐presenting function or cell–cell interactions.^103^

In addition to cell‐to‐cell interactions with adaptive immune cells, ILC3 have the capacity to modulate adaptive immune responses by the release of soluble mediators in the local tissue microenvironment. In this regard both gut NKp46^+^ and LTi‐like ILC3 are an important source of granulocyte–macrophage colony stimulating factor (GM‐CSF) under steady‐state conditions. Microbiota‐dependent IL‐1*β* production by intestinal macrophages activates ILC3 to secrete GM‐CSF, which in turn modulates mononuclear phagocyte function to promote local regulatory T‐cell differentiation and reinforce tolerance.^104^ ILC3 also express multiple proteins that endow them with the capacity to regulate B‐cell responses. ILC3 resident in Peyer's patches and intestinal lymphoid structures play key roles in the regulation of IgA production in the intestinal tract, via lymphotoxin‐dependent mechanisms.^41^, ^105^, ^106^, ^107^ LTi‐like ILC3 express both membrane‐bound lymphotoxin heterotrimers (LT*α*_1_ *β*_2_) as well as soluble lymphotoxin *α* homotrimers (LT*α*_3_), which control T‐independent and T‐dependent IgA responses, respectively.^105^, ^106^ Human and mouse splenic ILC3 also stimulate innate‐like B‐cell IgG production through CD40 interactions, production of BAFF/APRIL and via the Notch ligand Delta‐like 1.^108^, ^109^ Taken together ILC3 demonstrate increasingly expanding roles in modulating adaptive immune responses.

## ILC3 in inflammation and infection

In addition to maintaining tissue homeostasis ILC3 are an important source of cytokines that act to control mucosal infections. In the context of infection and inflammation, IL‐22 production by ILC3 is induced by cytokine signals derived from bacterial‐sensing myeloid cells, such as IL‐23 and IL‐1*β*.^73^, ^83^, ^96^, ^97^, ^110^ CX_3_CR_1_^+^ mononuclear phagocytes are a dominant source of IL‐23 during infection, which stimulates optimal ILC3 IL‐22 production.^111^ Conversely, ILC3 cytokine production is negatively regulated by epithelially derived cytokines such as thymic stromal lymphopoietin and IL‐25, which act to suppress IL‐22 production.^112^, ^113^ Therefore, signals from multiple intestinal resident cells combine to fine‐tune IL‐22 production by ILC3.

Extensive research has demonstrated protective roles of ILC3 during infection by using the bacterial pathogen *Citrobacter rodentium*, a model of intestinal inflammation and clinically relevant enteropathic *Escherichia coli* infection. ILC3‐derived IL‐22 is required for survival and immunity to *C. rodentium* infection in immunocompromised mice, whereas in the presence of adaptive immunity ILC3‐derived IL‐22 is required to prevent morbidity in the early phase of infection before the generation of a bacteria‐specific T‐cell response.^27^, ^114^, ^115^, ^116^, ^117^ As both subsets of ILC3 produce IL‐22 in the intestine the relative contribution of these two subsets to anti‐bacterial immunity has proven controversial. Initial studies proposed that NCR^+^ ILC3 are important during *C. rodentium* infection in mice with partial or total immunodeficiency.^27^, ^51^, ^91^, ^117^ However, NKp46^+^ ILC3 are dispensable for the control of *C. rodentium* infection in the presence of T cells.^84^, ^118^ In contrast, lack of total ILC3 results in impaired control of infection, suggesting that LTi‐like ILC3 may be sufficient for immunity to *C. rodentium*.^118^ In line with this, CD4^+^ LTi‐like ILC3 have been reported to be a dominant source of IL‐22 during the early stages of *C. rodentium* infection.^97^ Nonetheless, NKp46^+^ ILC3 may have other important non‐redundant roles during *C. rodentium* infection, as infected mice lacking NKp46^+^ ILC3 exhibited severe caecal damage.^84^ In addition, intestinal ILC3‐derived IL‐22 may also contribute to immunity roles in a diverse range of clinically relevant infections in the gut including the nosocomial pathogen *Clostridium difficile*,^119^ rotavirus,^120^ the fungal pathogen *Candida albicans*,^121^ as well as gastrointestinal helminths.^78^ In contrast, some enteric pathogens have evolved to circumvent and exploit the ILC3 response to infection. For example, *Salmonella* exploit IL‐22‐dependent antimicrobial responses to compete with the commensal microbiota and colonize the inflamed gut.^122^

ILC3 also contribute to local immunity and inflammation through the production of other cytokines. IL‐17A production by CCR6^+^ ILC3 is required for *Klebsiella pneumoniae* clearance and contributes to protection against systemic *Candida albicans* infection.^53^, ^123^ ILC3‐derived IL‐17A has further been associated with pathogenesis of obesity‐associated airway inflammation and psoriasis in mice and humans.^124^, ^125^, ^126^ Although relatively less characterized, ILC3 also secrete IL‐17F and a role for this cytokine in skin inflammation has also been suggested.^127^

Pro‐inflammatory cytokine production, such as IFN‐*γ* and tumour necrosis factor‐*α*, by NCR^+^ ILC3 and ‘ex‐ILC3’ may also contribute to protective immunity.^61^ However, chronic activation of ILC3 can result in disease due to uncontrolled inflammation. ILC3 drive colitis in an innate anti‐CD40 driven model of colitis and during *Helicobacter hepaticus* infection in an IL‐23‐dependent manner, which has been associated with production of GM‐CSF and IFN‐*γ* by pro‐inflammatory ILC3.^71^, ^118^, ^128^, ^129^ Neonatal ILC3 can also mediate intestinal pathology in response to transgenic over‐expression of IL‐23 through the release of IL‐17A, IL‐22, IFN‐*γ* and GM‐CSF.^130^ In line with a disease‐causing role of ILC3, Crohn's disease patients exhibit increased frequency of NCR^−^ (NKp44^−^ CD56^−^) CCR6^+^ ILC3 and have elevated levels of IL‐17A.^131^ Hence, in contrast to their protective and homeostatic roles, ILC3 may also take on pro‐inflammatory roles and contribute to tissue damage and inflammation during disease. Further studies are required to fully define the signals and environmental cues that regulate the pro‐ versus anti‐inflammatory phenotypes of ILC3 subsets.

## Concluding remarks

Group 3 ILC play central roles in lymphoid organogenesis, orchestration of adaptive immunity, regulation of peripheral tolerance and as effector cells in the context of immunity and inflammation. Recent studies have elegantly delineated the developmental and epigenetic networks that dictate ILC3 function and identified novel phenotypes that expand the spectrum of functions ascribed to ILC3 in mucosal barrier tissues. However, challenges remain in resolving the relevance of ILC3 heterogeneity and incorporating recently identified novel transcriptional and metabolic states in the context of current nomenclature. A more nuanced understanding of ILC3 subset‐specific roles in intestinal immunity will aid the development of targeted therapeutic interventions aimed at maintaining beneficial homeostatic ILC3 functions, while neutralizing pro‐inflammatory ILC3 pathways that contribute to the onset or progression of tissue inflammation.

## Disclosure

The authors have no competing interests to declare.

## References

[1] Klose CS , Artis D . Innate lymphoid cells as regulators of immunity, inflammation and tissue homeostasis. Nat Immunol 2016; 17:765–74. 27328006 10.1038/ni.3489

[2] Walker JA , Barlow JL , McKenzie AN . Innate lymphoid cells – how did we miss them? Nat Rev Immunol 2013; 13:75–87. 23292121 10.1038/nri3349

[3] Spits H , Artis D , Colonna M , Diefenbach A , Di Santo JP , Eberl G *et al* Innate lymphoid cells – a proposal for uniform nomenclature. Nat Rev Immunol 2013; 13:145–9. 23348417 10.1038/nri3365

[4] Diefenbach A , Colonna M , Koyasu S . Development, differentiation, and diversity of innate lymphoid cells. Immunity 2014; 41:354–65. 25238093 10.1016/j.immuni.2014.09.005 PMC4171710

[5] Klose CS , Flach M , Mohle L , Rogell L , Hoyler T , Ebert K *et al* Differentiation of type 1 ILCs from a common progenitor to all helper‐like innate lymphoid cell lineages. Cell 2014; 157:340–56. 24725403 10.1016/j.cell.2014.03.030

[6] Constantinides MG , Gudjonson H , McDonald BD , Ishizuka IE , Verhoef PA , Dinner AR *et al* PLZF expression maps the early stages of ILC1 lineage development. Proc Natl Acad Sci USA 2015; 112:5123–8. 25838284 10.1073/pnas.1423244112 PMC4413309

[7] Fuchs A , Vermi W , Lee JS , Lonardi S , Gilfillan S , Newberry RD *et al* Intraepithelial type 1 innate lymphoid cells are a unique subset of IL‐12‐ and IL‐15‐responsive IFN‐*γ*‐producing cells. Immunity 2013; 38:769–81. 23453631 10.1016/j.immuni.2013.02.010 PMC3634355

[8] Sonnenberg GF , Fouser LA , Artis D . Border patrol: regulation of immunity, inflammation and tissue homeostasis at barrier surfaces by IL‐22. Nat Immunol 2011; 12:383–90. 21502992 10.1038/ni.2025

[9] Serafini N , Vosshenrich CA , Di Santo JP . Transcriptional regulation of innate lymphoid cell fate. Nat Rev Immunol 2015; 15:415–28. 26065585 10.1038/nri3855

[10] Ishizuka IE , Constantinides MG , Gudjonson H , Bendelac A . The innate lymphoid cell precursor. Annu Rev Immunol 2016; 34:299–316. 27168240 10.1146/annurev-immunol-041015-055549

[11] Constantinides MG , McDonald BD , Verhoef PA , Bendelac A . A committed precursor to innate lymphoid cells. Nature 2014; 508:397–401. 24509713 10.1038/nature13047 PMC4003507

[12] Chea S , Schmutz S , Berthault C , Perchet T , Petit M , Burlen‐Defranoux O *et al* Single‐cell gene expression analyses reveal heterogeneous responsiveness of fetal innate lymphoid progenitors to notch signaling. Cell Rep 2016; 14:1500–16. 26832410 10.1016/j.celrep.2016.01.015

[13] Ishizuka IE , Chea S , Gudjonson H , Constantinides MG , Dinner AR , Bendelac A *et al* Single‐cell analysis defines the divergence between the innate lymphoid cell lineage and lymphoid tissue‐inducer cell lineage. Nat Immunol 2016; 17:269–76. 26779601 10.1038/ni.3344 PMC4755916

[14] Cherrier M , Sawa S , Eberl G . Notch, Id2, and ROR*γ*t sequentially orchestrate the fetal development of lymphoid tissue inducer cells. J Exp Med 2012; 209:729–40. 22430492 10.1084/jem.20111594 PMC3328368

[15] Satoh‐Takayama N , Lesjean‐Pottier S , Vieira P , Sawa S , Eberl G , Vosshenrich CA *et al* IL‐7 and IL‐15 independently program the differentiation of intestinal CD3‐NKp46^+^ cell subsets from Id2‐dependent precursors. J Exp Med 2010; 207:273–80. 20142427 10.1084/jem.20092029 PMC2822619

[16] Serafini N , Klein Wolterink RG , Satoh‐Takayama N , Xu W , Vosshenrich CA , Hendriks RW *et al* Gata3 drives development of ROR*γ*t^+^ group 3 innate lymphoid cells. J Exp Med 2014; 211:199–208. 24419270 10.1084/jem.20131038 PMC3920560

[17] Yagi R , Zhong C , Northrup DL , Yu F , Bouladoux N , Spencer S *et al* The transcription factor GATA3 is critical for the development of all IL‐7R*α*‐expressing innate lymphoid cells. Immunity 2014; 40:378–88. 24631153 10.1016/j.immuni.2014.01.012 PMC4026797

[18] Mielke LA , Groom JR , Rankin LC , Seillet C , Masson F , Putoczki T *et al* TCF‐1 controls ILC2 and NKp46^+^ROR*γ*t^+^ innate lymphocyte differentiation and protection in intestinal inflammation. J Immunol 2013; 191:4383–91. 24038093 10.4049/jimmunol.1301228

[19] Seillet C , Rankin LC , Groom JR , Mielke LA , Tellier J , Chopin M *et al* Nfil3 is required for the development of all innate lymphoid cell subsets. J Exp Med 2014; 211:1733–40. 25092873 10.1084/jem.20140145 PMC4144736

[20] Hoyler T , Klose CS , Souabni A , Turqueti‐Neves A , Pfeifer D , Rawlins EL *et al* The transcription factor GATA‐3 controls cell fate and maintenance of type 2 innate lymphoid cells. Immunity 2012; 37:634–48. 23063333 10.1016/j.immuni.2012.06.020 PMC3662874

[21] Yang Q , Monticelli LA , Saenz SA , Chi AW , Sonnenberg GF , Tang J *et al* T cell factor 1 is required for group 2 innate lymphoid cell generation. Immunity 2013; 38:694–704. 23601684 10.1016/j.immuni.2012.12.003 PMC4029843

[22] Zook EC , Kee BL . Development of innate lymphoid cells. Nat Immunol 2016; 17:775–82. 27328007 10.1038/ni.3481

[23] Yu Y , Tsang JC , Wang C , Clare S , Wang J , Chen X *et al* Single‐cell RNA‐seq identifies a PD‐1hi ILC progenitor and defines its developmental pathway. Nature 2016; 539:102–6. 27749818 10.1038/nature20105

[24] Eberl G , Marmon S , Sunshine MJ , Rennert PD , Choi Y , Littman DR . An essential function for the nuclear receptor ROR*γ*(t) in the generation of fetal lymphoid tissue inducer cells. Nat Immunol 2004; 5:64–73. 14691482 10.1038/ni1022

[25] Luci C , Reynders A , Ivanov II , Cognet C , Chiche L , Chasson L *et al* Influence of the transcription factor ROR*γ*t on the development of NKp46^+^ cell populations in gut and skin. Nat Immunol 2009; 10:75–82. 19029904 10.1038/ni.1681

[26] Sanos SL , Bui VL , Mortha A , Oberle K , Heners C , Johner C *et al* ROR*γ*t and commensal microflora are required for the differentiation of mucosal interleukin 22‐producing NKp46^+^ cells. Nat Immunol 2009; 10:83–91. 19029903 10.1038/ni.1684 PMC4217274

[27] Satoh‐Takayama N , Vosshenrich CA , Lesjean‐Pottier S , Sawa S , Lochner M , Rattis F *et al* Microbial flora drives interleukin 22 production in intestinal NKp46^+^ cells that provide innate mucosal immune defense. Immunity 2008; 29:958–70. 19084435 10.1016/j.immuni.2008.11.001

[28] Possot C , Schmutz S , Chea S , Boucontet L , Louise A , Cumano A *et al* Notch signaling is necessary for adult, but not fetal, development of ROR*γ*t^+^ innate lymphoid cells. Nat Immunol 2011; 12:949–58. 21909092 10.1038/ni.2105

[29] Montaldo E , Teixeira‐Alves LG , Glatzer T , Durek P , Stervbo U , Hamann W *et al* Human ROR*γ*t^+^CD34^+^ cells are lineage‐specified progenitors of group 3 ROR*γ*t^+^ innate lymphoid cells. Immunity 2014; 41:988–1000. 25500367 10.1016/j.immuni.2014.11.010

[30] Sawa S , Cherrier M , Lochner M , Satoh‐Takayama N , Fehling HJ , Langa F *et al* Lineage relationship analysis of ROR*γ*t^+^ innate lymphoid cells. Science 2010; 330:665–9. 20929731 10.1126/science.1194597

[31] Scoville SD , Mundy‐Bosse BL , Zhang MH , Chen L , Zhang X , Keller KA *et al* A progenitor cell expressing transcription factor ROR*γ*t generates all human innate lymphoid cell subsets. Immunity 2016; 44:1140–50. 27178467 10.1016/j.immuni.2016.04.007 PMC4893782

[32] Onder L , Danuser R , Scandella E , Firner S , Chai Q , Hehlgans T *et al* Endothelial cell‐specific lymphotoxin‐beta receptor signaling is critical for lymph node and high endothelial venule formation. J Exp Med 2013; 210:465–73. 23420877 10.1084/jem.20121462 PMC3600902

[33] Mebius RE , Rennert P , Weissman IL . Developing lymph nodes collect CD4^+^CD3^–^ LT*β* ^+^ cells that can differentiate to APC, NK cells, and follicular cells but not T or B cells. Immunity 1997; 7:493–504. 9354470 10.1016/s1074-7613(00)80371-4

[34] van de Pavert SA , Mebius RE . New insights into the development of lymphoid tissues. Nat Rev Immunol 2010; 10:664–74. 20706277 10.1038/nri2832

[35] Kim MY , McConnell FM , Gaspal FM , White A , Glanville SH , Bekiaris V *et al* Function of CD4^+^CD3^–^ cells in relation to B‐ and T‐zone stroma in spleen. Blood 2007; 109:1602–10. 17018858 10.1182/blood-2006-04-018465

[36] Eberl G . Inducible lymphoid tissues in the adult gut: recapitulation of a fetal developmental pathway? Nat Rev Immunol 2005; 5:413–20. 15841100 10.1038/nri1600

[37] Lindemans CA , Calafiore M , Mertelsmann AM , O'Connor MH , Dudakov JA , Jenq RR *et al* Interleukin‐22 promotes intestinal‐stem‐cell‐mediated epithelial regeneration. Nature 2015; 528:560–4. 26649819 10.1038/nature16460 PMC4720437

[38] Aparicio‐Domingo P , Romera‐Hernandez M , Karrich JJ , Cornelissen F , Papazian N , Lindenbergh‐Kortleve DJ *et al* Type 3 innate lymphoid cells maintain intestinal epithelial stem cells after tissue damage. J Exp Med 2015; 212:1783–91. 26392223 10.1084/jem.20150318 PMC4612094

[39] Hanash AM , Dudakov JA , Hua G , O'Connor MH , Young LF , Singer NV *et al* Interleukin‐22 protects intestinal stem cells from immune‐mediated tissue damage and regulates sensitivity to graft versus host disease. Immunity 2012; 37:339–50. 22921121 10.1016/j.immuni.2012.05.028 PMC3477611

[40] Bouskra D , Brezillon C , Berard M , Werts C , Varona R , Boneca IG *et al* Lymphoid tissue genesis induced by commensals through NOD1 regulates intestinal homeostasis. Nature 2008; 456:507–10. 18987631 10.1038/nature07450

[41] Tsuji M , Suzuki K , Kitamura H , Maruya M , Kinoshita K , Ivanov II *et al* Requirement for lymphoid tissue‐inducer cells in isolated follicle formation and T cell‐independent immunoglobulin A generation in the gut. Immunity 2008; 29:261–71. 18656387 10.1016/j.immuni.2008.05.014

[42] Gomez de Aguero M , Ganal‐Vonarburg SC , Fuhrer T , Rupp S , Uchimura Y , Li H *et al* The maternal microbiota drives early postnatal innate immune development. Science 2016; 351:1296–302. 26989247 10.1126/science.aad2571

[43] van de Pavert SA , Ferreira M , Domingues RG , Ribeiro H , Molenaar R , Moreira‐Santos L *et al* Maternal retinoids control type 3 innate lymphoid cells and set the offspring immunity. Nature 2014; 508:123–7. 24670648 10.1038/nature13158 PMC4932833

[44] Goverse G , Labao‐Almeida C , Ferreira M , Molenaar R , Wahlen S , Konijn T *et al* Vitamin A controls the presence of ROR*γ* ^+^ innate lymphoid cells and lymphoid tissue in the small intestine. J Immunol 2016; 196:5148–55. 27183576 10.4049/jimmunol.1501106

[45] Spencer SP , Wilhelm C , Yang Q , Hall JA , Bouladoux N , Boyd A *et al* Adaptation of innate lymphoid cells to a micronutrient deficiency promotes type 2 barrier immunity. Science 2014; 343:432–7. 24458645 10.1126/science.1247606 PMC4313730

[46] Kiss EA , Vonarbourg C , Kopfmann S , Hobeika E , Finke D , Esser C *et al* Natural aryl hydrocarbon receptor ligands control organogenesis of intestinal lymphoid follicles. Science 2011; 334:1561–5. 22033518 10.1126/science.1214914

[47] Qiu J , Heller JJ , Guo X , Chen ZM , Fish K , Fu YX *et al* The aryl hydrocarbon receptor regulates gut immunity through modulation of innate lymphoid cells. Immunity 2012; 36:92–104. 22177117 10.1016/j.immuni.2011.11.011 PMC3268875

[48] Lee JS , Cella M , McDonald KG , Garlanda C , Kennedy GD , Nukaya M *et al* AHR drives the development of gut ILC22 cells and postnatal lymphoid tissues via pathways dependent on and independent of Notch. Nat Immunol 2012; 13:144–51. 10.1038/ni.2187 PMC3468413 22101730

[49] Qiu J , Guo X , Chen ZM , He L , Sonnenberg GF , Artis D *et al* Group 3 innate lymphoid cells inhibit T‐cell‐mediated intestinal inflammation through aryl hydrocarbon receptor signaling and regulation of microflora. Immunity 2013; 39:386–99. 23954130 10.1016/j.immuni.2013.08.002 PMC3884586

[50] Chea S , Perchet T , Petit M , Verrier T , Guy‐Grand D , Banchi EG *et al* Notch signaling in group 3 innate lymphoid cells modulates their plasticity. Sci Signal 2016; 9:ra45. 27141929 10.1126/scisignal.aaf2223

[51] Rankin LC , Groom JR , Chopin M , Herold MJ , Walker JA , Mielke LA *et al* The transcription factor T‐bet is essential for the development of NKp46^+^ innate lymphocytes via the Notch pathway. Nat Immunol 2013; 14:389–95. 23455676 10.1038/ni.2545 PMC4076532

[52] Gasteiger G , Fan X , Dikiy S , Lee SY , Rudensky AY . Tissue residency of innate lymphoid cells in lymphoid and nonlymphoid organs. Science 2015; 350:981–5. 26472762 10.1126/science.aac9593 PMC4720139

[53] Withers DR , Hepworth MR , Wang X , Mackley EC , Halford EE , Dutton EE *et al* Transient inhibition of ROR‐gammat therapeutically limits intestinal inflammation by reducing TH17 cells and preserving group 3 innate lymphoid cells. Nat Med 2016; 22:319–23. 26878233 10.1038/nm.4046 PMC4948756

[54] Kloverpris HN , Kazer SW , Mjosberg J , Mabuka JM , Wellmann A , Ndhlovu Z *et al* Innate lymphoid cells are depleted irreversibly during acute HIV‐1 infection in the absence of viral suppression. Immunity 2016; 44:391–405. 26850658 10.1016/j.immuni.2016.01.006 PMC6836297

[55] Hepworth MR , Fung TC , Masur SH , Kelsen JR , McConnell FM , Dubrot J *et al* Immune tolerance. Group 3 innate lymphoid cells mediate intestinal selection of commensal bacteria‐specific CD4^+^ T cells. Science 2015; 348:1031–5. 25908663 10.1126/science.aaa4812 PMC4449822

[56] Robinette ML , Fuchs A , Cortez VS , Lee JS , Wang Y , Durum SK *et al* Transcriptional programs define molecular characteristics of innate lymphoid cell classes and subsets. Nat Immunol 2015; 16:306–17. 25621825 10.1038/ni.3094 PMC4372143

[57] Bjorklund AK , Forkel M , Picelli S , Konya V , Theorell J , Friberg D *et al* The heterogeneity of human CD127^+^ innate lymphoid cells revealed by single‐cell RNA sequencing. Nat Immunol 2016; 17:451–60. 26878113 10.1038/ni.3368

[58] Koues OI , Collins PL , Cella M , Robinette ML , Porter SI , Pyfrom SC *et al* Distinct gene regulatory pathways for human innate versus adaptive lymphoid cells. Cell 2016; 165:1134–46. 27156452 10.1016/j.cell.2016.04.014 PMC4874868

[59] Shih HY , Sciume G , Mikami Y , Guo L , Sun HW , Brooks SR *et al* Developmental acquisition of regulomes underlies innate lymphoid cell functionality. Cell 2016; 165:1120–33. 27156451 10.1016/j.cell.2016.04.029 PMC4874839

[60] Gury‐BenAri M , Thaiss CA , Serafini N , Winter DR , Giladi A , Lara‐Astiaso D *et al* The spectrum and regulatory landscape of intestinal innate lymphoid cells are shaped by the microbiome. Cell 2016; 166:1231–46 e13. 27545347 10.1016/j.cell.2016.07.043

[61] Klose CS , Kiss EA , Schwierzeck V , Ebert K , Hoyler T , d'Hargues Y *et al* A T‐bet gradient controls the fate and function of CCR6‐ROR*γ*t^+^ innate lymphoid cells. Nature 2013; 494:261–5. 23334414 10.1038/nature11813

[62] Vitenshtein A , Charpak‐Amikam Y , Yamin R , Bauman Y , Isaacson B , Stein N *et al* NK cell recognition of *Candida glabrata* through binding of NKp46 and NCR1 to fungal ligands Epa1, Epa6, and Epa7. Cell Host Microbe 2016; 20:527–34. 27736647 10.1016/j.chom.2016.09.008 PMC5492882

[63] Glatzer T , Killig M , Meisig J , Ommert I , Luetke‐Eversloh M , Babic M *et al* ROR*γ*t^+^ innate lymphoid cells acquire a proinflammatory program upon engagement of the activating receptor NKp44. Immunity 2013; 38:1223–35. 23791642 10.1016/j.immuni.2013.05.013

[64] Johnson H , Scorrano L , Korsmeyer SJ , Ley TJ . Cell death induced by granzyme C. Blood 2003; 101:3093–101. 12515723 10.1182/blood-2002-08-2485

[65] Cortez VS , Colonna M . Diversity and function of group 1 innate lymphoid cells. Immunol Lett 2016; 179:19–24. 27394699 10.1016/j.imlet.2016.07.005 PMC5658203

[66] Veiga‐Fernandes H , Coles MC , Foster KE , Patel A , Williams A , Natarajan D *et al* Tyrosine kinase receptor RET is a key regulator of Peyer's patch organogenesis. Nature 2007; 446:547–51. 17322904 10.1038/nature05597

[67] Ibiza S , Garcia‐Cassani B , Ribeiro H , Carvalho T , Almeida L , Marques R *et al* Glial‐cell‐derived neuroregulators control type 3 innate lymphoid cells and gut defence. Nature 2016; 535:440–3. 27409807 10.1038/nature18644 PMC4962913

[68] Olivier BJ , Cailotto C , van der Vliet J , Knippenberg M , Greuter MJ , Hilbers FW *et al* Vagal innervation is required for the formation of tertiary lymphoid tissue in colitis. Eur J Immunol 2016; 46:2467–80. 27457277 10.1002/eji.201646370

[69] Weiss JM , Bilate AM , Gobert M , Ding Y , Curotto de Lafaille MA , Parkhurst CN *et al* Neuropilin 1 is expressed on thymus‐derived natural regulatory T cells, but not mucosa‐generated induced Foxp3^+^ T reg cells. J Exp Med 2012; 209:1723–42, S1. 22966001 10.1084/jem.20120914 PMC3457733

[70] Yadav M , Louvet C , Davini D , Gardner JM , Martinez‐Llordella M , Bailey‐Bucktrout S *et al* Neuropilin‐1 distinguishes natural and inducible regulatory T cells among regulatory T cell subsets *in vivo* . J Exp Med 2012; 209:1713–22, S1‐19. 22966003 10.1084/jem.20120822 PMC3457729

[71] Vonarbourg C , Mortha A , Bui VL , Hernandez PP , Kiss EA , Hoyler T *et al* Regulated expression of nuclear receptor RORgammat confers distinct functional fates to NK cell receptor‐expressing ROR*γ*t^+^ innate lymphocytes. Immunity 2010; 33:736–51. 21093318 10.1016/j.immuni.2010.10.017 PMC3042726

[72] Bernink JH , Peters CP , Munneke M , te Velde AA , Meijer SL , Weijer K *et al* Human type 1 innate lymphoid cells accumulate in inflamed mucosal tissues. Nat Immunol 2013; 14:221–9. 23334791 10.1038/ni.2534

[73] Bernink JH , Krabbendam L , Germar K , de Jong E , Gronke K , Kofoed‐Nielsen M *et al* Interleukin‐12 and ‐23 control plasticity of CD127^+^ Group 1 and Group 3 innate lymphoid cells in the intestinal lamina propria. Immunity 2015; 43:146–60. 26187413 10.1016/j.immuni.2015.06.019

[74] Verrier T , Satoh‐Takayama N , Serafini N , Marie S , Di Santo JP , Vosshenrich CA . Phenotypic and functional plasticity of murine intestinal NKp46^+^ Group 3 innate lymphoid cells. J Immunol 2016; 196:4731–8. 27183613 10.4049/jimmunol.1502673

[75] Viant C , Rankin LC , Girard‐Madoux MJ , Seillet C , Shi W , Smyth MJ *et al* Transforming growth factor‐*β* and Notch ligands act as opposing environmental cues in regulating the plasticity of type 3 innate lymphoid cells. Sci Signal 2016; 9:ra46. 27141930 10.1126/scisignal.aaf2176

[76] Hepworth MR , Monticelli LA , Fung TC , Ziegler CG , Grunberg S , Sinha R *et al* Innate lymphoid cells regulate CD4^+^ T‐cell responses to intestinal commensal bacteria. Nature 2013; 498:113–7. 23698371 10.1038/nature12240 PMC3699860

[77] Mukherjee S , Hooper LV . Antimicrobial defense of the intestine. Immunity 2015; 42:28–39. 25607457 10.1016/j.immuni.2014.12.028

[78] Turner JE , Stockinger B , Helmby H . IL‐22 mediates goblet cell hyperplasia and worm expulsion in intestinal helminth infection. PLoS Pathog 2013; 9:e1003698. 24130494 10.1371/journal.ppat.1003698 PMC3795034

[79] Gulhane M , Murray L , Lourie R , Tong H , Sheng YH , Wang R *et al* High fat diets induce colonic epithelial cell stress and inflammation that is reversed by IL‐22. Sci Rep 2016; 6:28990. 27350069 10.1038/srep28990 PMC4924095

[80] Shih VF , Cox J , Kljavin NM , Dengler HS , Reichelt M , Kumar P *et al* Homeostatic IL‐23 receptor signaling limits Th17 response through IL‐22‐mediated containment of commensal microbiota. Proc Natl Acad Sci USA 2014; 111:13942–7. 25201978 10.1073/pnas.1323852111 PMC4183330

[81] Ivanov II , Atarashi K , Manel N , Brodie EL , Shima T , Karaoz U *et al* Induction of intestinal Th17 cells by segmented filamentous bacteria. Cell 2009; 139:485–98. 19836068 10.1016/j.cell.2009.09.033 PMC2796826

[82] Zenewicz LA , Yin X , Wang G , Elinav E , Hao L , Zhao L *et al* IL‐22 deficiency alters colonic microbiota to be transmissible and colitogenic. J Immunol 2013; 190:5306–12. 23585682 10.4049/jimmunol.1300016 PMC3646987

[83] Sano T , Huang W , Hall JA , Yang Y , Chen A , Gavzy SJ *et al* An IL‐23R/IL‐22 circuit regulates epithelial serum amyloid A to promote local effector Th17 responses. Cell 2015; 163:381–93. 26411290 10.1016/j.cell.2015.08.061 PMC4621768

[84] Rankin LC , Girard‐Madoux MJ , Seillet C , Mielke LA , Kerdiles Y , Fenis A *et al* Complementarity and redundancy of IL‐22‐producing innate lymphoid cells. Nat Immunol 2016; 17:179–86. 26595889 10.1038/ni.3332 PMC4720992

[85] Pham TA , Clare S , Goulding D , Arasteh JM , Stares MD , Browne HP *et al* Epithelial IL‐22RA1‐mediated fucosylation promotes intestinal colonization resistance to an opportunistic pathogen. Cell Host Microbe 2014; 16:504–16. 25263220 10.1016/j.chom.2014.08.017 PMC4190086

[86] Pickard JM , Maurice CF , Kinnebrew MA , Abt MC , Schenten D , Golovkina TV *et al* Rapid fucosylation of intestinal epithelium sustains host‐commensal symbiosis in sickness. Nature 2014; 514:638–41. 25274297 10.1038/nature13823 PMC4214913

[87] Goto Y , Obata T , Kunisawa J , Sato S , Ivanov II , Lamichhane A *et al* Innate lymphoid cells regulate intestinal epithelial cell glycosylation. Science 2014; 345:1254009. 25214634 10.1126/science.1254009 PMC4774895

[88] Sonnenberg GF , Monticelli LA , Alenghat T , Fung TC , Hutnick NA , Kunisawa J *et al* Innate lymphoid cells promote anatomical containment of lymphoid‐resident commensal bacteria. Science 2012; 336:1321–5. 22674331 10.1126/science.1222551 PMC3659421

[89] Fung TC , Bessman NJ , Hepworth MR , Kumar N , Shibata N , Kobuley D *et al* Lymphoid‐tissue‐resident commensal bacteria promote members of the IL‐10 cytokine family to establish mutualism. Immunity 2016; 44:634–46. 26982365 10.1016/j.immuni.2016.02.019 PMC4845739

[90] Obata T , Goto Y , Kunisawa J , Sato S , Sakamoto M , Setoyama H *et al* Indigenous opportunistic bacteria inhabit mammalian gut‐associated lymphoid tissues and share a mucosal antibody‐mediated symbiosis. Proc Natl Acad Sci USA 2010; 107:7419–24. 20360558 10.1073/pnas.1001061107 PMC2867693

[91] Satoh‐Takayama N , Serafini N , Verrier T , Rekiki A , Renauld JC , Frankel G *et al* The chemokine receptor CXCR6 controls the functional topography of interleukin‐22 producing intestinal innate lymphoid cells. Immunity 2014; 41:776–88. 25456160 10.1016/j.immuni.2014.10.007

[92] Chea S , Possot C , Perchet T , Petit M , Cumano A , Golub R *et al* CXCR6 expression is important for retention and circulation of ILC precursors. Mediators Inflamm 2015; 2015:368427. 26494947 10.1155/2015/368427 PMC4606447

[93] Hepworth MR , Sonnenberg GF . Regulation of the adaptive immune system by innate lymphoid cells. Curr Opin Immunol 2014; 27:75–82. 24594491 10.1016/j.coi.2014.01.013 PMC3979357

[94] Mackley EC , Houston S , Marriott CL , Halford EE , Lucas B , Cerovic V *et al* CCR7‐dependent trafficking of ROR*γ* ^+^ ILCs creates a unique microenvironment within mucosal draining lymph nodes. Nat Commun 2015; 6:5862. 25575242 10.1038/ncomms6862 PMC4354100

[95] von Burg N , Chappaz S , Baerenwaldt A , Horvath E , Bose Dasgupta S , Ashok D *et al* Activated group 3 innate lymphoid cells promote T‐cell‐mediated immune responses. Proc Natl Acad Sci USA 2014; 111:12835–40. 25136120 10.1073/pnas.1406908111 PMC4156721

[96] Goto Y , Panea C , Nakato G , Cebula A , Lee C , Diez MG *et al* Segmented filamentous bacteria antigens presented by intestinal dendritic cells drive mucosal Th17 cell differentiation. Immunity 2014; 40:594–607. 24684957 10.1016/j.immuni.2014.03.005 PMC4084624

[97] Sonnenberg GF , Monticelli LA , Elloso MM , Fouser LA , Artis D *et al* CD4^+^ lymphoid tissue‐inducer cells promote innate immunity in the gut. Immunity 2011; 34:122–34. 21194981 10.1016/j.immuni.2010.12.009 PMC3035987

[98] Withers DR , Gaspal FM , Mackley EC , Marriott CL , Ross EA , Desanti GE *et al* Cutting edge: Lymphoid tissue inducer cells maintain memory CD4 T cells within secondary lymphoid tissue. J Immunol 2012; 189:2094–8. 22855716 10.4049/jimmunol.1201639 PMC3442242

[99] Gaspal FM , Kim MY , McConnell FM , Raykundalia C , Bekiaris V , Lane PJ . Mice deficient in OX40 and CD30 signals lack memory antibody responses because of deficient CD4 T cell memory. J Immunol 2005; 174:3891–6. 15778343 10.4049/jimmunol.174.7.3891

[100] Kim MY , Gaspal FM , Wiggett HE , McConnell FM , Gulbranson‐Judge A , Raykundalia C *et al* CD4^+^CD3^–^ accessory cells costimulate primed CD4 T cells through OX40 and CD30 at sites where T cells collaborate with B cells. Immunity 2003; 18:643–54. 12753741 10.1016/s1074-7613(03)00110-9

[101] Korn LL , Thomas HL , Hubbeling HG , Spencer SP , Sinha R , Simkins HM *et al* Conventional CD4^+^ T cells regulate IL‐22‐producing intestinal innate lymphoid cells. Mucosal Immunol 2014; 7:1045–57. 24448096 10.1038/mi.2013.121 PMC4107180

[102] Saez de Guinoa J , Jimeno R , Farhadi N , Jervis PJ , Cox LR , Besra GS *et al* CD1d‐mediated activation of group 3 innate lymphoid cells drives IL‐22 production. EMBO Rep 2016; doi: 10.15252/embr.201642412. 10.15252/embr.201642412 PMC5210076 27799287

[103] Bank U , Deiser K , Finke D , Hammerling GJ , Arnold B , Schuler T . Cutting edge: innate lymphoid cells suppress homeostatic T cell expansion in neonatal mice. J Immunol 2016; 196:3532–6. 26983785 10.4049/jimmunol.1501643

[104] Mortha A , Chudnovskiy A , Hashimoto D , Bogunovic M , Spencer SP , Belkaid Y *et al* Microbiota‐dependent crosstalk between macrophages and ILC3 promotes intestinal homeostasis. Science 2014; 343:1249288. 24625929 10.1126/science.1249288 PMC4291125

[105] Reboldi A , Arnon TI , Rodda LB , Atakilit A , Sheppard D , Cyster JG . IgA production requires B cell interaction with subepithelial dendritic cells in Peyer's patches. Science 2016; 352:aaf4822. 27174992 10.1126/science.aaf4822 PMC4890166

[106] Kruglov AA , Grivennikov SI , Kuprash DV , Winsauer C , Prepens S , Seleznik GM *et al* Nonredundant function of soluble LT*α*3 produced by innate lymphoid cells in intestinal homeostasis. Science 2013; 342:1243–6. 24311691 10.1126/science.1243364

[107] Macpherson AJ , Koller Y , McCoy KD . The bilateral responsiveness between intestinal microbes and IgA. Trends Immunol 2015; 36:460–70. 26169256 10.1016/j.it.2015.06.006

[108] Cella M , Otero K , Colonna M . Expansion of human NK‐22 cells with IL‐7, IL‐2, and IL‐1*β* reveals intrinsic functional plasticity. Proc Natl Acad Sci USA 2010; 107:10961–6. 20534450 10.1073/pnas.1005641107 PMC2890739

[109] Magri G , Miyajima M , Bascones S , Mortha A , Puga I , Cassis L *et al* Innate lymphoid cells integrate stromal and immunological signals to enhance antibody production by splenic marginal zone B cells. Nat Immunol 2014; 15:354–64. 24562309 10.1038/ni.2830 PMC4005806

[110] Seo SU , Kuffa P , Kitamoto S , Nagao‐Kitamoto H , Rousseau J , Kim YG *et al* Intestinal macrophages arising from CCR2^+^ monocytes control pathogen infection by activating innate lymphoid cells. Nat Commun 2015; 6:8010. 26269452 10.1038/ncomms9010 PMC4536571

[111] Longman RS , Diehl GE , Victorio DA , Huh JR , Galan C , Miraldi ER *et al* CX(3)CR1^+^ mononuclear phagocytes support colitis‐associated innate lymphoid cell production of IL‐22. J Exp Med 2014; 211:1571–83. 25024136 10.1084/jem.20140678 PMC4113938

[112] Giacomin PR , Moy RH , Noti M , Osborne LC , Siracusa MC , Alenghat T *et al* Epithelial‐intrinsic IKK*α* expression regulates group 3 innate lymphoid cell responses and antibacterial immunity. J Exp Med 2015; 212:1513–28. 26371187 10.1084/jem.20141831 PMC4577836

[113] Sawa S , Lochner M , Satoh‐Takayama N , Dulauroy S , Berard M , Kleinschek M *et al* ROR*γ*t^+^ innate lymphoid cells regulate intestinal homeostasis by integrating negative signals from the symbiotic microbiota. Nat Immunol 2011; 12:320–6. 21336274 10.1038/ni.2002

[114] Zheng Y , Valdez PA , Danilenko DM , Hu Y , Sa SM , Gong Q *et al* Interleukin‐22 mediates early host defense against attaching and effacing bacterial pathogens. Nat Med 2008; 14:282–9. 18264109 10.1038/nm1720

[115] Guo X , Qiu J , Tu T , Yang X , Deng L , Anders RA *et al* Induction of innate lymphoid cell‐derived interleukin‐22 by the transcription factor STAT3 mediates protection against intestinal infection. Immunity 2014; 40:25–39. 24412612 10.1016/j.immuni.2013.10.021 PMC3919552

[116] Basu R , O'Quinn DB , Silberger DJ , Schoeb TR , Fouser L , Ouyang W *et al* Th22 cells are an important source of IL‐22 for host protection against enteropathogenic bacteria. Immunity 2012; 37:1061–75. 23200827 10.1016/j.immuni.2012.08.024 PMC3678257

[117] Cella M , Fuchs A , Vermi W , Facchetti F , Otero K , Lennerz JK *et al* A human natural killer cell subset provides an innate source of IL‐22 for mucosal immunity. Nature 2009; 457:722–5. 18978771 10.1038/nature07537 PMC3772687

[118] Song C , Lee JS , Gilfillan S , Robinette ML , Newberry RD , Stappenbeck TS *et al* Unique and redundant functions of NKp46^+^ ILC3s in models of intestinal inflammation. J Exp Med 2015; 212:1869–82. 26458769 10.1084/jem.20151403 PMC4612098

[119] Abt MC , Lewis BB , Caballero S , Xiong H , Carter RA , Susac B *et al* Innate immune defenses mediated by two ILC subsets are critical for protection against acute *Clostridium difficile* infection. Cell Host Microbe 2015; 18:27–37. 26159718 10.1016/j.chom.2015.06.011 PMC4537644

[120] Hernandez PP , Mahlakoiv T , Yang I , Schwierzeck V , Nguyen N , Guendel F *et al* Interferon‐*λ* and interleukin 22 act synergistically for the induction of interferon‐stimulated genes and control of rotavirus infection. Nat Immunol 2015; 16:698–707. 26006013 10.1038/ni.3180 PMC4589158

[121] Zelante T , Iannitti RG , Cunha C , De Luca A , Giovannini G , Pieraccini G *et al* Tryptophan catabolites from microbiota engage aryl hydrocarbon receptor and balance mucosal reactivity via interleukin‐22. Immunity 2013; 39:372–85. 23973224 10.1016/j.immuni.2013.08.003

[122] Behnsen J , Jellbauer S , Wong CP , Edwards RA , George MD , Ouyang W *et al* The cytokine IL‐22 promotes pathogen colonization by suppressing related commensal bacteria. Immunity 2014; 40:262–73. 24508234 10.1016/j.immuni.2014.01.003 PMC3964146

[123] Xiong H , Keith JW , Samilo DW , Carter RA , Leiner IM , Pamer EG . Innate Lymphocyte/Ly6C^hi^ monocyte crosstalk promotes *Klebsiella pneumoniae* clearance. Cell 2016; 165:679–89. 27040495 10.1016/j.cell.2016.03.017 PMC4842125

[124] Kim HY , Lee HJ , Chang YJ , Pichavant M , Shore SA , Fitzgerald KA *et al* Interleukin‐17‐producing innate lymphoid cells and the NLRP3 inflammasome facilitate obesity‐associated airway hyperreactivity. Nat Med 2014; 20:54–61. 24336249 10.1038/nm.3423 PMC3912313

[125] Villanova F , Flutter B , Tosi I , Grys K , Sreeneebus H , Perera GK *et al* Characterization of innate lymphoid cells in human skin and blood demonstrates increase of NKp44^+^ ILC3 in psoriasis. J Invest Dermatol 2014; 134:984–91. 24352038 10.1038/jid.2013.477 PMC3961476

[126] Dyring‐Andersen B , Geisler C , Agerbeck C , Lauritsen JP , Gudjonsdottir SD , Skov L *et al* Increased number and frequency of group 3 innate lymphoid cells in nonlesional psoriatic skin. Br J Dermatol 2014; 170:609–16. 24125475 10.1111/bjd.12658

[127] Li Z , Hodgkinson T , Gothard EJ , Boroumand S , Lamb R , Cummins I *et al* Epidermal Notch1 recruits ROR*γ* ^+^ group 3 innate lymphoid cells to orchestrate normal skin repair. Nat Commun 2016; 7:11394. 27099134 10.1038/ncomms11394 PMC4844683

[128] Buonocore S , Ahern PP , Uhlig HH , Ivanov II , Littman DR , Maloy KJ *et al* Innate lymphoid cells drive interleukin‐23‐dependent innate intestinal pathology. Nature 2010; 464:1371–5. 20393462 10.1038/nature08949 PMC3796764

[129] Pearson C , Thornton EE , McKenzie B , Schaupp AL , Huskens N , Griseri T *et al* ILC3 GM‐CSF production and mobilisation orchestrate acute intestinal inflammation. Elife 2016; 5:e10066. 26780670 10.7554/eLife.10066 PMC4733039

[130] Chen L , He Z , Slinger E , Bongers G , Lapenda TL , Pacer ME *et al* IL‐23 activates innate lymphoid cells to promote neonatal intestinal pathology. Mucosal Immunol 2015; 8:390–402. 25160819 10.1038/mi.2014.77 PMC4326561

[131] Geremia A , Arancibia‐Carcamo CV , Fleming MP , Rust N , Singh B , Mortensen NJ *et al* IL‐23‐responsive innate lymphoid cells are increased in inflammatory bowel disease. J Exp Med 2011; 208:1127–33. 21576383 10.1084/jem.20101712 PMC3173242

